# Bermuda 2.0: reflections from Santa Cruz

**DOI:** 10.1093/gigascience/giw003

**Published:** 2016-12-29

**Authors:** Jenny Reardon, Rachel A. Ankeny, Jenny Bangham, Katherine W. Darling, Stephen Hilgartner, Kathryn Maxson Jones, Beth Shapiro, Hallam Stevens, The Genomic Open workshop group

**Affiliations:** 1 Department of Sociology, University of California Santa Cruz, 1156 High Street, Santa Cruz, CA, 95064, USA; 2 Department of History. Floor 3, Room 11, Napier Building, North Terrace Campus, University of Adelaide, Adelaide, SA 5005, Australia; 3 Department of History and Philosophy of Science, University of Cambridge, Free School Lane, Cambridge, CB2 3RH, UK; 4 Department of Science and Technology Studies, Cornell University, 303 Morrill Hall, Cornell University, Ithaca, NY 14853, USA; 5 Program in History of Science, Department of History, Princeton University, 129 Dickinson Hall, Princeton, NJ 08544-1017, USA; 6 Department of Ecology and Evolutionary Biology, University of California Santa Cruz, 1156 High Street, Santa Cruz, CA 95064, USA; 7 School of Humanities and Social Sciences, Nanyang Technological University, 14 Nanyang Drive #05-07, 637332, Singapore

**Keywords:** Data, Sharing, Bermuda Principles, Openness

## Abstract

In February 1996, the genome community met in Bermuda to formulate principles for circulating genomic data. Although it is now 20 years since the Bermuda Principles were formulated, they continue to play a central role in shaping genomic and data-sharing practices. However, since 1996, “openness” has become an increasingly complex issue. This commentary seeks to articulate three core challenges data-sharing faces today.

## Background

**Fig. 1:**
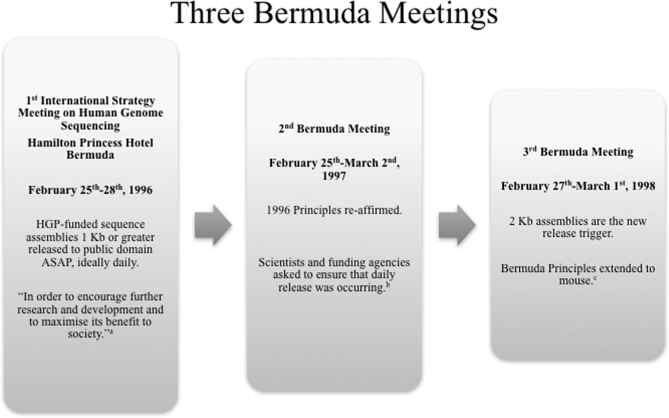
Three “Bermuda Meetings” were held in 1996^a^, 1997^b^, and 1998^c^. At each subsequent meeting the principles for data sharing were affirmed, extended, updated, and refined.

In February 1996, leaders in genome science convened in Bermuda and penned principles for circulating genomic data that endure today [1]. The story of the Bermuda Principles and the commitment to daily sharing of DNA sequences prior to publication has become one of the dominant narratives of the Human Genome Project (HGP). Motivated in part by an attempt to keep the human genome sequence in the public domain, the Bermuda Principles inaugurated a commitment to openness at the heart of the new field of genomics [Fig 1]. Since 1996, however, the issues surrounding “openness” have become increasingly complex, raising new questions about the meaning of openness itself, and how and why it should be enacted and enforced.

Two decades after the meeting in Bermuda, on November 18, 2015, some original members of the Bermuda meetings, along with other genome scientists and social scientists, gathered at University of California Santa Cruz to reflect on what ‘open genomics’ means in the context of the post-HGP conditions: the exponential growth of genomic data, the centrality of private funding, and commitment to the right of privacy [2]. The hypothesis at Santa Cruz was that revisiting the historic Bermuda Principles would clarify what is at stake in today's decisions about how and whether to share data, with whom, and on what platforms. The participants articulated three core challenges that suggest ways to frame the ethical, political, and technical dilemmas that lie ahead.

## Challenge one: what is data?

In 1996, the ‘data’ that occupied participants’ attention were the nucleotide sequences needed to create a single human reference sequence. Today, however, the forms of relevant data are proliferating, creating new puzzles for clinicians, genome scientists, epidemiologists, and potential patients and research participants. We have expanded our capabilities from analyzing and interpreting one individual's data to exponential numbers of people and exponential amounts of data. These derive not just from sequences, but also from other ‘omics’ data, such as metabolomics, metagenomics, proteomics, epigenomics and exposomics, which is now increasingly linked to socio-economic, behavioral, genealogical, clinical, and GIS data. Despite manifest differences between different types of data, the Bermuda Principles are often invoked as a touchstone for the “right” approach to use and reuse.

However, the uses and value of these data - and proper structures for their governance - are often far from clear. Practitioners within different subfields collect, process, clean, report, and analyze data in different ways. What *counts* as data thus often depends on specific disciplinary norms, standards, and modes of valuation. Data collected via automated, high-throughput techniques may be valued differently by experimenters, publishers and regulators than curated data or data coded through long-term fieldwork.

**Fig. 2:**
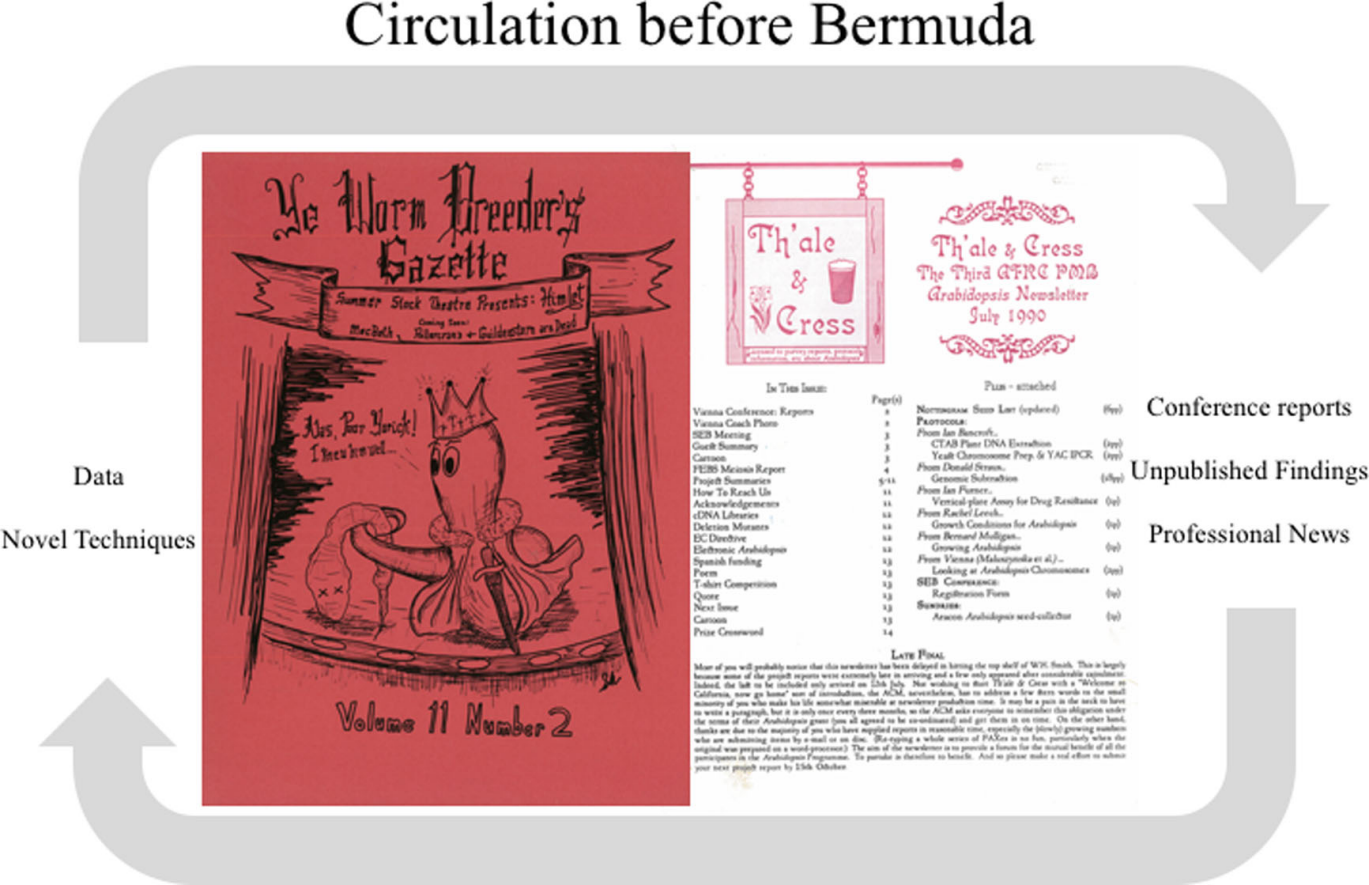
Covers of biology community newsletters; here, Arabidopsis *Newsletter* (1990) and *Worm Breeder's Gazette* (1990). These, like *Drosophila Information Service* and many others (see [3] for a partial list), helped to adjudicate community membership and mediate sharing and ownership. They typically communicated technical innovations, nomenclatures, community news and lists of which (living) stocks could be obtained from what laboratories. Cover images courtesy of Department of Genetics Library, University of Cambridge.

Furthermore, practices for creating valuable data in small communities often differ from those in very large ones. Before and during the early phases of the HGP, model organism communities (in particular, those studying *Caenorhabditis elegans* and *Drosophila*) developed varied norms and practices for collecting, curating, and communicating data. At Santa Cruz, Jenny Bangham, Robert Kuhn, and Bob Waterston discussed some early tools used for sharing information and assigning credit, such as the newsletters Drosophila Information Service and The Worm Breeder's Gazette [3]. Even in such close-knit communities, pre-publication ‘sharing’ occurred within carefully managed networks and systems of trust and credit [Fig. 2].

Today genomic data are no longer created solely within the confines of model organism communities. Rather, data are often donated by individuals with interests in how they are used. How then do scientists make decisions about data's value when there is no community to ensure quality control (e.g., for species that are not model organisms)? Without the guidance of community norms, how should we decide when data are good enough to share?

## Challenge two: what is sharing?

Alongside new criteria and practices for creating valuable data, we require new standards and practices for sharing. When the HGP began in the early 1990s, its funders decided that scientists needed to share the incomplete data they produced; but, more precisely what needed to be shared, with whom, when and how was a matter of debate. These were the problems that the Bermuda meetings (including those in 1997 and 1998) attempted to address. The enduring challenge today is that ‘sharing’ is value-heavy, but conceptually thin. Sharing is an almost universally embraced value. The concept of sharing, however, does not capture the technical complexity or specificity of what it means to deposit, store, transfer, exchange, transport, or interconnect genomic data and health information in a digitally networked world [4].

Clarifying the goals of data sharing is harder today than it was two decades ago. Making large amounts of data widely available for a long period of time and re-usable by third-parties involves substantial human and infrastructural resources. Who will support the storage, upload, curation and publication of ever expanding quantities of data? And who will ensure the privacy and interests of patients and research participants? What are the promises and limits of technical solutions? What methods will engender trust? And how will credit be allocated?

In Santa Cruz, Stephen Hilgartner called for greater nuance in how we describe the governance of data throughout the process of producing, collecting, and exchanging them, both before and after publication. He suggested conceptualizing various “data regimes” for guiding projects in biomedicine [5]. Data, he argued, exist within governing structures that delineate the roles of funders, scientists, laboratories, universities, data storage infrastructure, algorithms, medical industries, human subjects, and institutional review boards. Each of these entities is endowed with rights, responsibilities, and privileges for accessing and controlling data. Describing such rights and responsibilities explicitly will help us to clarify the goals, tradeoffs, and beneficiaries of data sharing.

## Challenge three: what is a public good?

It may seem that the Bermuda Principles presented a clear vision of the public good: open data shared immediately with the scientific community. Bermuda embraced the idea that the HGP sequence data were a self-evident public good. Yet, at the Santa Cruz meeting Kathryn Maxson and Rachel Ankeny offered an analysis of the Bermuda meetings showing that the issues were not clear cut [6]. Bermuda participants from European countries and Japan raised concerns that a 24-hour release would impede the ability of government-funded scientists to make good on their research investments through patents. For them, private pharmaceutical development represented a public good. Many at the Bermuda meetings—not just those from Europe and Japan—viewed open data and commercial products as mutually reinforcing. Indeed, recent economic analyses show how genomic sequences in the public domain spurred more commercialization and for-profit drug development than did the restricted data from Craig Venter's Celera Genomics [7].

Today, as universities, governments and companies collaborate and compete to create the platforms that make data flow, we can no longer rely on a simplistic distinction between public and private to conceptualize good data governance. Acquiring the ever-escalating resources needed for generating data leads scientists to seek funding from varied sources. Indeed, large sums of money often no longer raise suspicions, but garner esteem. If we can no longer rely on the public/private boundary to delineate good approaches to sharing data, we are left with a final pressing puzzle: how do we ensure that data leads to knowledge and the public good?

Many of the tests and treatments arising from genomic data have to date been extremely expensive and have not seen wide clinical utility [8]. How do we ensure equitable distribution of the benefits? While genomics cannot solve the ‘social ills’ of healthcare systems, data governance cannot ignore the fact that today's -omic data are collected and used within inequitable and fragmented healthcare infrastructures, particularly in the US [9]. A policy of open data will not guarantee that everyone has equal access or benefits. We thus face the challenge of creating not just an open, but also a *just* approach to sharing biomedical data [10].

## Toward ‘Good’ genomic science

The moral grounds that solidified during the HGP, complex as they were, no longer provide adequate guidance. At Santa Cruz, we updated the received views about the Bermuda Meetings, transforming the principles of 1996 into key challenges for 2016 and beyond. The Santa Cruz challenges show us that we need a better understanding of the actual practices and stakes involved in data sharing. We must clarify what we mean when we talk about genomic data and “the public good.” Understanding how value is created through specific flows of data will lay the groundwork for more engaged deliberations about the benefits and drawbacks of various sharing regimes. Developing robust agreements about data governance in the postgenomic era requires creating experimental spaces and cross-disciplinary dialogues such as those at Santa Cruz.

### Abbreviations

HGP Human Genome Project.

### Competing interests

The authors and collaborators have no competing interests to declare.

### Funding

Financial support for the Genomic Open workshop was provided by the Science and Justice Research Center and the UC Santa Cruz Genomics Institute.

### Authors’ contributions

JR led the writing of this essay and drafted the first draft with KD. HS, SH, and JR led the drafting of the shortened version. All authors contributed to the writing of the final version. JB, KMJ and HS created the infographics.
